# Predictive parameters and model for extubation outcome in pediatric patients

**DOI:** 10.3389/fped.2023.1151068

**Published:** 2023-04-03

**Authors:** Kan Charernjiratragul, Kantara Saelim, Kanokpan Ruangnapa, Kantisa Sirianansopa, Pharsai Prasertsan, Wanaporn Anuntaseree

**Affiliations:** Division of Pulmonology and Critical Care Medicine, Department of Pediatrics, Faculty of Medicine, Prince of Songkla University, Songkhla, Thailand

**Keywords:** mechanical ventilation, morbidity, pediatric patients, extubation outcome, predictive model

## Abstract

**Background:**

Prolonged mechanical ventilation is associated with significant morbidity in critically ill pediatric patients. In addition, extubation failure and deteriorating respiratory status after extubation contribute to increased morbidity. Well-prepared weaning procedures and accurate identification of at-risk patients using multimodal ventilator parameters are warranted to improve patient outcomes. This study aimed to identify and assess the diagnostic accuracy of single parameters and to develop a model that can help predict extubation outcomes.

**Materials and methods:**

This prospective observational study was conducted at a university hospital between January 2021 and April 2022. Patients aged 1 month to 15 years who were intubated for more than 12 h and deemed clinically ready for extubation were enrolled. A weaning process with a spontaneous breathing trial (SBT), with or without minimal setting, was employed. The ventilator and patient parameters during the weaning period at 0, 30, and 120 min and right before extubation were recorded and analyzed.

**Results:**

A total of 188 eligible patients were extubated during the study. Of them, 45 (23.9%) patients required respiratory support escalation within 48 h. Of 45, 13 (6.9%) were reintubated. The predictors of respiratory support escalation consisted of a nonminimal-setting SBT [odds ratio (OR) 2.2 (1.1, 4.6), *P* = 0.03], >3 ventilator days [OR 2.4 (1.2, 4.9), *P* = 0.02], occlusion pressure (P0.1) at 30 min ≥0.9 cmH_2_O [OR 2.3 (1.1, 4.9), *P* = 0.03], and exhaled tidal volume per kg at 120 min ≤8 ml/kg [OR 2.2 (1.1, 4.6), *P* = 0.03]; all of these predictors had an area under the curve (AUC) of 0.72. A predictive scoring system to determine the probability of respiratory support escalation was developed using a nomogram.

**Conclusion:**

The proposed predictive model, which integrated both patient and ventilator parameters, showed a modest performance level (AUC 0.72); however, it could facilitate the process of patient care.

## 1. Introduction

Mechanical ventilation is an important life-support system in critically ill patients. However, prolonged intubation can lead to several complications such as nosocomial infections, airway trauma, and increased healthcare utilization (1). The trend toward optimizing outcomes involves minimizing the duration of mechanical ventilation. The weaning process and accurate determination of extubation readiness are imperative for all ventilated patients.

To date, there is no definite standard weaning method or extubation guidance for the pediatric population. An earlier study assessed the effects of the parameters of both pre-extubation patients and ventilators; however, it did not demonstrate any cutoff value or useful clinical implications for bedside care (2). Several parameters have been assessed individually, such as respiratory rate (RR), arterial blood gas, tidal volume (Vt) and maximum inspiratory pressure (3). Other parameters were assessed as collective indexes such as the rapid shallow breathing index (RSBI), pressure index, and CROP index to predict successful extubation (4–8). However, these parameters were predominantly used in research and not in a clinical setting (9). Moreover, some of these parameters have proven helpful in adults but are not generally used in children (10). The occlusion pressure (P0.1) is the decrease in airway pressure 100 ms after occluded inspiration (11). P0.1 is a reliable measure of respiratory drive, as it represents an involuntary reaction to the mechanical load during the first millisecond and is independent of patient effort (12). An automated P0.1 measurement is available on modern pediatric ventilators, which can demonstrate how well they correlate with the reference values of P0.1. However, no study has investigated the role of P0.1 in facilitating the weaning and extubation processes in pediatric patients.

The factors effecting extubation outcome may include many aspects namely respiratory muscle function, pulmonary function, sedation level, risk of airway edema, and systemic condition. Previously, predictors of extubation outcomes were investigated as a single parameter. However, single parameters have a limited ability to predict the extubation outcome. Moreover, available data regarding the performance and cutoff values related to the previously mentioned parameters in pediatric patients are limited. Recently, a predictive model was introduced to enhance the ability of personalized factors to predict outcomes. However, the predictive model for extubation outcomes has been studied in preterm newborns and adults (13, 14) but not in the pediatric population. At present, noninvasive respiratory support is commonly used to facilitate extubation in patients. Unfavorable outcomes after extubation include the need for reintubation and deteriorating respiratory status. Therefore, our study aimed to assess the diagnostic accuracy of each parameter and evaluate a predictive model that combined the predictive parameters to predict respiratory support escalation after extubation.

## 2. Methods

This prospective observational study was conducted at the pediatric intensive care unit (PICU) of a single referral university-affiliated center in Southern Thailand. Children aged 1 month to 15 years who were mechanically ventilated for more than 12 h in the PICU between January 2021 and April 2022 were enrolled. Patients with large endotracheal tube leakage (>50%) were excluded because of invalid exhalation ventilator parameters. This study was approved by the institutional review board of Faculty Medicine, Prince of Songkla University. Informed consent was obtained from the patients’ parents or guardians when the patient's clinical condition was stable and before the weaning process.

### 2.1. Weaning protocol

When the eligible patients were cardiopulmonary stable, the attending physician initiated the weaning process. The ventilator mode was adjusted to the weaning setting, that is, the spontaneous mode with pressure support (PS). Thereafter, the attending physician adjusted the ventilator parameters according to the patient's status. Minimal-setting spontaneous breathing trial (SBT) was defined as patients receiving PS with positive end-expiratory pressure (PEEP) 3–5 cmH_2_O, minimal PS based on endotracheal tube size (15), and fractional oxygen (FiO_2_) < 0.6. If patients could not tolerate the SBT, the attending physician adjusted the ventilator setting to higher than that of the minimal-setting SBT, classified as nonminimal-setting SBT. After quiet breathing was achieved, the ventilator variables consisted of P0.1, exhaled Vt (Vte), RR, PS, PEEP, and FiO_2_. Delta Vte was defined by the change in Vte from the initiation of SBT to 120 min after SBT to measure the decrease in tidal volume after performing SBT. Patient parameters such as oxygen saturation and vital signs were recorded at 0, 30, and 120 min and right before extubation. All ventilator variables were derived using Servo-I™ version 8.0. In addition, the corrected rapid shallow breathing index (cRSBI) was calculated by dividing the RR by Vte per the patient's ideal body weight. The standard protocol of our institutional extubation guidelines was applied to all patients to ensure good preparation and proper management (Supplementary Figure S1). All data recordings were performed in a double-blind fashion by a nurse who was not involved in caring for the participating patients. After extubation, the attending physician provided standard care and initiated respiratory support according to the patient's decision considering his/her respiratory status. Extubation outcomes included the type of respiratory support immediately and at 1 and 48 h after extubation. Furthermore, PICU outcomes were assessed and recorded.

### 2.2. Definitions

Extubation outcome was assessed 48 h after extubation. Respiratory support escalation was indicated by an increasing amount of oxygen flow or FiO_2_ in a noninvasive ventilator (NIV) or heated humidified high-flow nasal cannula (HFNC) and extubation failure. Extubation failure was defined as a requirement for reintubation. Patients with uneventful extubation were classified into the liberation respiratory support group. The cause of escalation support was recorded and patients who required escalation of respiratory support, due to stridor and respiratory distress, were classified into the upper airway obstruction (UAO) group for the subgroup analysis.

### 2.3. Statistical analysis

The R program (version 4.2.0, R Foundation for Statistical Computing, Vienna, Austria) was used for data analysis. Categorical variables were presented as frequencies and percentages and were compared using either the chi-square test or Fisher's exact test. Nonparametric continuous variables were presented as median [interquartile range (IQR)] and were investigated using the dependent *t*-test as well as the rank sum test. In addition, multivariate analyses were conducted to identify potential factors associated with uneventful extubation. Variables with a *P* value of <0.2 were included in the multivariate model. Variable selection for each multivariate model was based on backward elimination. A *P* value <0.05 was considered statistically significant. The optimal cutoff points for P0.1, cRSBI, and Vt were defined according to the Youden method. The area under the receiver operating characteristic curve and the calculated area under the curve (AUC) were used to determine the discriminative ability of the cutoff points. A predictive scoring system for the probability of respiratory support escalation was developed using a nomogram. The model was validated using the bootstrap resampling method.

## 3. Results

In this study, 188 mechanically ventilated pediatric patients were enrolled. The median age at the time of PICU admission and ventilator days was 1.7 years (IQR 0.4, 5.6) and 3 days (IQR 1, 6.2), respectively. The predominant indication for intubation was postoperative status (45.7%), followed by pneumonia (15.4%) and congestive heart failure (12.2%) (Table 1). Forty-five patients required respiratory support escalation after extubation within 48 h, 13 of whom (6.9%) required reintubation. The causes of respiratory support escalation were upper airway obstruction (63%) and respiratory distress due to causes other than UAO (37%).

**Table 1:** Baseline characteristics of enrolled participants.

Patient characteristics	Escalation support	Liberation support	Total	*P* value
	*n* = 45 (%)	*n* = 143 (%)	*n* = 188	
**Age**^a^ **(years)**	1.6 (0.4, 4.3)	1.9 (0.4, 5.9)	1.7 (0.4, 5.6)	0.606
**Male**	23 (51.1)	91 (63.6)	114 (60.6)	0.185
**Failure to thrive**	24 (53.3)	54 (37)	78 (4.15)	0.09
**Intubation indication**				0.071
Upper airway obstruction	5 (11.1)	6 (4.2)	11 (5.9)	
Pneumonia	8 (17.8)	21 (14.7)	29 (15.4)	
Lower airway obstruction	0 (0)	2 (1.4)	2 (1.1)	
Congestive heart failure	9 (20)	14 (9.8)	23 (12.2)	
Alteration of consciousness	5 (11.1)	16 (11.2)	21 (11.2)	
Postoperative	13 (28.9)	73 (51)	86 (45.7)	
Shock	5 (11.1)	8 (5.6)	13 (6.9)	
Other	0 (0)	3 (2.1)	3 (1.6)	
**Type of underlying disease**				0.677
Respiratory	7 (15.6)	24 (16.8)	31 (16.5)	
Cardiovascular	23 (51.1)	57 (39.9)	80 (42.6)	
Neurological	9 (20)	39 (27.3)	48 (25.5)	
Gastrointestinal	4 (8.9)	9 (6.3)	13 (6.9)	
Nephrological	0 (0)	1 (0.7)	1 (0.5)	
Hematologic	2 (4.4)	13 (9.1)	15 (8)	
**Days of ventilator use** ^a^	4 (2, 9)	2 (1, 6)	3 (1, 6.2)	0.004

^a^ Median (interquartile range).

There was no difference in patient characteristics and underlying diseases between the respiratory support escalation and liberation groups (Table 1). The support escalation group was associated with significantly more ventilator days than the respiratory support liberation group [4 (2, 9) vs. 2 (1, 6), *P* = 0.004]. Significant differences in ventilator parameters before extubation between the groups are shown in Table 2. Other parameters are listed in Supplementary Table S1. Data on the initial type of respiratory support are presented in Supplementary Table S2.

**Table 2:** The significantly different ventilator parameters between the study groups.

Ventilator parameters	Escalation support	Liberation support	*P* value
	*n* = 45 (%)	*n* = 143 (%)	
Nonminimal-setting SBT	24 (53.3)	46 (32.2)	0.02
PEEP > 5 cmH_2_O	18 (40)	31 (21.7)	0.02
Vti/kg at 120 min^a^	8.6 (5.8, 11.2)	9.5 (7.6, 12)	0.04
Vte/kg at 120 min^a^	7.2 (5.1, 10)	8.8 (7, 11.2)	0.01
Delta Vte/kg^a^	−0.8 (−2.2, 0.7)	0.2 (−0.9, 1.5)	0.03
P0.1 at 30 min^a^	1 (0.8, 1.4)	0.8 (0.5, 1.4)	0.06

SBT, spontaneous breathing trial; Vti, inspired tidal volume; Vte, exhaled tidal volume; Delta Vte/kg, exhaled tidal volume change between 120 and 0 min; P0.1, occlusion pressure.

^a^ Median (interquartile range).

### 3.1. Primary outcome

P0.1 and cRSBI were not significantly different between both groups in all five measurement time periods during weaning. P0.1 at 30 min after extubation showed an almost statistically significant trend for a higher median in the escalation support group (*P* = 0.06). The optimal cutoff point of P0.1 at 30 min to predict support escalation was determined to be >0.9, with a sensitivity of 69%, specificity of 52%, and an AUC of 0.59. The optimal cutoff point for cRSBI at the beginning of the SBT was 7 breaths/min/ml/kg, with a sensitivity of 69%, specificity of 29%, and AUC of 0.54. The delta Vte change of <0 ml between 120 and 0 min had a sensitivity of 38%, specificity of 46%, and an AUC of 0.61. Patients with UAO after extubation were excluded from the subgroup analysis. The overall performance of the remaining parameters was similar to that of the entire study population (Supplementary Table S3). Details of the adjusted predictors in the final multivariate analysis are shown in Table 3.

**Table 3:** Multivariate analysis of predictors of respiratory support escalation.

Predictive factors	OR (95% CI)	*P* value
Nonminimal-setting SBT	2.2 (1.1, 4.6)	0.03
>3 ventilator days	2.4 (1.2, 4.9)	0.02
P0.1 at 30 min ≥0.9 cmH_2_O	2.3 (1.1, 4.9)	0.03
Vte/kg at 120 min ≤8 ml/kg	2.2 (1.1, 4.6)	0.03

OR, odds ratio; CI, confidence interval; SBT, spontaneous breathing trial; P0.1, occlusion pressure; Vte, exhaled tidal volume.

### 3.2. Secondary outcome

**Figure 1:**
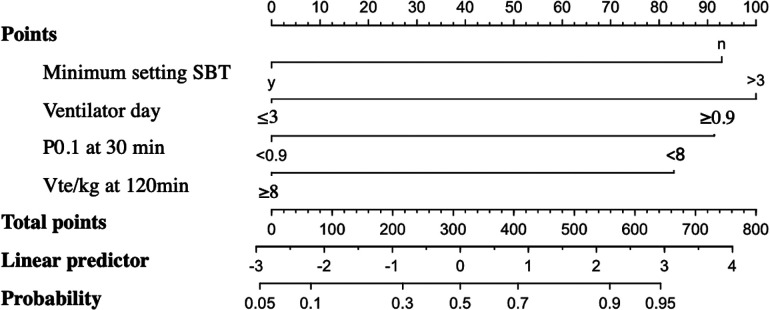
Nomogram for predicting the probability of support escalation based on the summation of the scores for each factor. SBT with a minimal setting: yes = 0 points and no = 92 points. Ventilator days: ≤3 = 0 points and >3 = 100 points. P0.1 at 30 min: <0.9 cm H_2_O = 0 points and ≥0.9 = 91 points. Vte/kg (exhaled tidal volume) at 120 min: >8 mL/kg = 0 points and <8 mL/kg = 83 points. To sum up the total points (range, 0–800), a line was drawn from the total point axis to the probability axis. SBT, spontaneous breathing trial; P0.1, occlusion pressure; Vte: exhaled tidal volume.

The predictive scoring system for the probability of respiratory support escalation was developed using a nomogram, which included four parameters that were significant after multivariate analysis. The score weights of the parameters varied from 83 to 100, whereas the total score ranged from 0 to 800 (Figure 1). Assessment of the model's performance indicated a moderate discriminative ability (AUC 0.72). The optimal cutoff point was 150 with a sensitivity of 71, and specificity of 60. Moreover, the model was applied to our study population and subgroup populations for internal validation; an error range of 3.3%–4.1% was found (Supplementary Figures S2–S4).

## 4. Discussion

At present, multimodal measures, such as standard weaning protocols, noninvasive ventilation, and HFNC, are used to facilitate successful extubation. Extubation outcomes, such as extubation failure and escalation of respiratory support after extubation, are relevant to the optimization of patient care. Krasinkiewicz et al. investigated respiratory support modalities and progression of patient outcomes after extubation and found that some patients required intensification of respiratory support from HFNC to NIV (16). The present study revealed that 45 of the 188 participants (23.9%) required escalation of respiratory support, and 6.9% of the enrolled participants needed reintubation. Generally, failed extubation rates vary between 3% and 22% depending on the underlying disease, illness severity, and indication for intubation (17). Most of the participants in this study were postoperative patients; hence, the lower rate of reintubation. This finding was similar to that reported by another study on a similar population (18). In addition, the initiation of a protocol-based weaning approach with increased elective use of noninvasive respiratory support after extubation also lowered the extubation failure rate.

This study identified a pragmatic approach to extubation outcome prognostication by employing both patient variables and ventilator indices. Consistent with the findings of a previous study, the duration of mechanical ventilation (in days) was found to be a strong predictor (2). P0.1 was used as a reflection of the patient's respiratory drive. Lower P0.1 values reflect a low respiratory drive, whereas higher P0.1 values signify a high central respiratory drive and respiratory effort indicating insufficient respiratory effort. Nevertheless, there are scant data about the cutoff point and utility of P0.1 in pediatric patients. Our study found an optimal P0.1 cutoff point of >0.9 cmH_2_O at 30 min, which showed high sensitivity in the prediction of respiratory support escalation. In contrast, Manczur et al. found a lower median P0.1 in the failed extubation group (*P* = 0.06) (19). This contradiction in the findings regarding the P0.1 values associated with extubation outcomes might be explained by the cause of respiratory failure. In our study, the most common cause was UAO; therefore, our clinical presentations were less likely to be associated with poor respiratory drive compared to those reported in other studies. Moreover, as per the study protocol, sedative medications were carefully adjusted to achieve the appropriate level.

RSBI is the ratio between RR and Vt (RR/Vt) and has been widely used in the adult population. The modification of the cRSBI, which is based on the patient's weight, was adjusted to suit the pediatric population. The threshold cutoff point of cRSBI at the time of SBT initiation was 7 breaths/min/ml/kg, which was comparable to the cutoff point found in a previous study (5). To our knowledge, the change in Vte during weaning has not been previously investigated. Decreasing the Vt over time during SBT increases the risk of respiratory support escalation after extubation. This finding indicated decreased lung compliance and respiratory muscle strength.

Although protocol-based weaning is well-established in adults, no pediatric studies have shown a clear superiority of the protocol-based approach over the physician's individualized decision (20). Earlier studies have used SBTs with various methods and reported inconsistent outcomes (21–23). In this study, patients who successfully passed 2 h of minimal-setting SBT had a lower risk of requiring respiratory support escalation.

Although multiple predictors were analyzed in this study, the accuracy of each weaning indicator exhibited a limited ability to predict escalation of respiratory support. Presently, predictive models utilizing combined parameters are increasing in popularity as tools of choice for both diagnosis and prognosis. However, no other study has explored this predictive model in a pediatric population. In the present study, the predictive model consisted of four feasible bedside indexes: nonminimal-setting SBT, >3 ventilator days, P0.1 at 30 min of ≥0.9 cmH_2_O, and Vte/kg at 120 min of ≤8 ml/kg. This predictive model showed a superior ability, compared with single parameters, and a moderate level of diagnostic accuracy (AUC, 0.72) for prognosticate escalation of care. The probability of escalating respiratory support according to this model could prove influential in optimizing patient care, for example, preparing and closely monitoring at-risk patients.

This study has some limitations. As the majority of the study population consisted of postoperative patients, the extubation failure rate was low. Moreover, parameters associated with UAO after extubation were not explored. However, after excluding patients with UAO, the subgroup analysis revealed unchanged outcomes. Furthermore, inhomogeneous indications for intubation could have affected the study outcomes. In light of these limitations, the generalizability of our study's results requires careful consideration. A large prospective study investigating the performance of predictive parameters is warranted. Moreover, the proposed predictive scoring system requires external validation.

## 5. Conclusion

A P0.1 value of >0.9 cmH_2_O at 30 min after SBT, corrected RSBI of >7 breaths/min/ml/kg, decreased Vt over 120 min of SBT, >3 ventilator days, and nonminimal-setting SBT were found to be risks for respiratory support escalation after extubation. The evaluated predictive model, which utilized parameters in combination, performed best when defining the probability of the need for support escalation after extubation.

## Data Availability

The raw data supporting the conclusions of this article will be made available by the authors, without undue reservation.

## References

[1] Ely EW Baker AM Dunagan DP Burke HL Smith AC Kelly PT Effect on the duration of mechanical ventilation of identifying patients capable of breathing spontaneously. N Engl J Med. (1996) 335:1864–9. 10.1056/NEJM199612193352502 8948561

[2] Laham JL Breheny PJ Rush A. Do clinical parameters predict first planned extubation outcome in the pediatric intensive care unit? J Intensive Care Med. (2015) 30:89–96. 10.1177/0885066613494338 23813884

[3] Ng P Tan HL Ma YJ Sultana R Long V Wong JJM Tests and indices predicting extubation failure in children: a systematic review and meta-analysis. Pulm Ther. (2023) 9:25–47. 10.1007/s41030-022-00204-w 36459328 PMC9931987

[4] Moura JCDS Gianfrancesco L Souza TH Hortencio TDR Nogueira RJN. Extubation in the pediatric intensive care unit: predictive methods. An integrative literature review. Rev Bras Ter Intensiva. (2021) 33:304–11. 10.5935/0103-507X.20210039 34231812 PMC8275073

[5] Thiagarajan RR Bratton SL Martin LD Brogan TV Taylor D. Predictors of successful extubation in children. Am J Respir Crit Care Med. (1999) 160:1562–6. 10.1164/ajrccm.160.5.9810036 10556121

[6] Khan N Brown A Venkataraman ST. Predictors of extubation success and failure in mechanically ventilated infants and children. Crit Care Med. (1996) 24:1568–79. 10.1097/00003246-199609000-00023 8797633

[7] Toida C Muguruma T Miyamoto M. Detection and validation of predictors of successful extubation in critically ill children. PLoS One. (2017) 12:e0189787. 10.1371/journal.pone.0189787 29253019 PMC5734724

[8] Venkataraman ST Khan N Brown A. Validation of predictors of extubation success and failure in mechanically ventilated infants and children. Crit Care Med. (2000) 28(8):2991–6. 10.1097/00003246-200008000-00051 10966284

[9] Newth CJ Venkataraman S Willson DF Meert KL Harrison R Dean JM Weaning and extubation readiness in pediatric patients. Pediatr Crit Care Med. (2009) 10:1–11. 10.1097/PCC.0b013e318193724d 19057432 PMC2849975

[10] Saikia B Kumar N Sreenivas V. Prediction of extubation failure in newborns, infants and children: brief report of a prospective (blinded) cohort study at a tertiary care paediatric centre in India. Springerplus. (2015) 4:827. 10.1186/s40064-015-1607-1 26753114 PMC4695462

[11] Telias I Damiani F Brochard L. The airway occlusion pressure (P0.1) to monitor respiratory drive during mechanical ventilation: increasing awareness of a not-so-new problem. Intensive Care Med. (2018) 44:1532–5. 10.1007/s00134-018-5045-8 29350241

[12] Ferreira FV Sugo EK Aragon DC Carmona F Carlotti APCP. Spontaneous breathing trial for prediction of extubation success in pediatric patients following congenital heart surgery: a randomized controlled trial. Pediatr Crit Care Med. (2019) 20:940–6. 10.1097/PCC.0000000000002006 31162372

[13] Sato R Hasegawa D Hamahata NT Narala S Nishida K Takahashi K The predictive value of airway occlusion pressure at 100 msec (P0.1) on successful weaning from mechanical ventilation: a systematic review and meta-analysis. J Crit Care. (2021) 63:124–32. 10.1016/j.jcrc.2020.09.030 33012587

[14] Baptistella AR Mantelli LM Matte L Carvalho MEDRU Fortunatti JA Costa IZ Prediction of extubation outcome in mechanically ventilated patients: development and validation of the extubation predictive score (ExPreS). PLoS One. (2021) 16:e0248868. 10.1371/journal.pone.0248868 33735250 PMC7971695

[15] Gupta D Greenberg RG Sharma A Natarajan G Cotten M Thomas R A predictive model for extubation readiness in extremely preterm infants. J Perinatol. (2019) 39:1663–9. 10.1038/s41372-019-0475-x 31455825

[16] Faustino EV Gedeit R Schwarz AJ Asaro LA Wypij D Curley MA Accuracy of an extubation readiness test in predicting successful extubation in children with acute respiratory failure from lower respiratory tract disease. Crit Care Med. (2017) 45:94–102. 10.1097/CCM.0000000000002024 27632676 PMC5541896

[17] Krasinkiewicz JM Friedman ML Slaven JE Tori AJ Lutfi R Abu-Sultaneh S. Progression of respiratory support following pediatric extubation. Pediatr Crit Care Med. (2020) 21:e1069–75. 10.1097/PCC.0000000000002520 32804740

[18] Kurachek SC Newth CJ Quasney MW Rice T Sachdeva RC Patel NR Extubation failure in pediatric intensive care: a multiple-center study of risk factors and outcomes. Crit Care Med. (2003) 31:2657–64. 10.1097/01.CCM.0000094228.90557.85 14605539

[19] Munshi FA Bukhari ZM Alshaikh H Saem Aldahar M Alsafrani T Elbehery M. Rapid shallow breathing index as a predictor of extubation outcomes in pediatric patients underwent cardiac surgeries at king faisal cardiac center. Cureus. (2020) 12:e8754. 10.7759/cureus.8754 32714692 PMC7377672

[20] Manczur TI Greenough A Pryor D Rafferty GF. Assessment of respiratory drive and muscle function in the pediatric intensive care unit and prediction of extubation failure. Pediatr Crit Care Med. (2000) 1:124–6. 10.1097/00130478-200010000-00006 12813262

[21] Elisa P Francesca C Marco P Davide V Laura Z Fabrizio Z Ventilation weaning and extubation readiness in children in pediatric intensive care unit: a review. Front Pediatr. (2022) 10:867739. 10.3389/fped.2022.867739 35433554 PMC9010786

[22] Ferguson LP Walsh BK Munhall D Arnold JH. A spontaneous breathing trial with pressure support overestimates readiness for extubation in children. Pediatr Crit Care Med. (2011) 12:e330–5. 10.1097/PCC.0b013e3182231220 21666529

[23] Farias JA Retta A Alía I Olazarri F Esteban A Golubicki A A comparison of two methods to perform a breathing trial before extubation in pediatric intensive care patients. Intensive Care Med. (2001) 27:1649–54. 10.1007/s001340101035 11685307

