# Supplementary material for: Predictive parameters and model for extubation outcome in pediatric patients
Source: Front Pediatr. 2023 Apr 3;11:1151068. doi: 10.3389/fped.2023.1151068 (PMC10106763; doi:10.3389/fped.2023.1151068)
Supplement: Supplementary file 1 [file Datasheet1.docx]

Supplementary Material

Predictive Parameters and Model for Extubation Outcome in Pediatric Patients

Kan Charernjiratragul^1^, Kantara Saelim^2*^ , Kanokpan Ruangnapa^3^, Kantisa Sirianansopa^4^, Pharsai Prasertsan^5^, Wanaporn Anuntaseree^6^

*** Correspondence:** Kantara Saelim: [kantara.s@psu.ac.th](mailto:kantara.s@psu.ac.th)

# Supplementary Figures and Tables

## Supplementary Figures


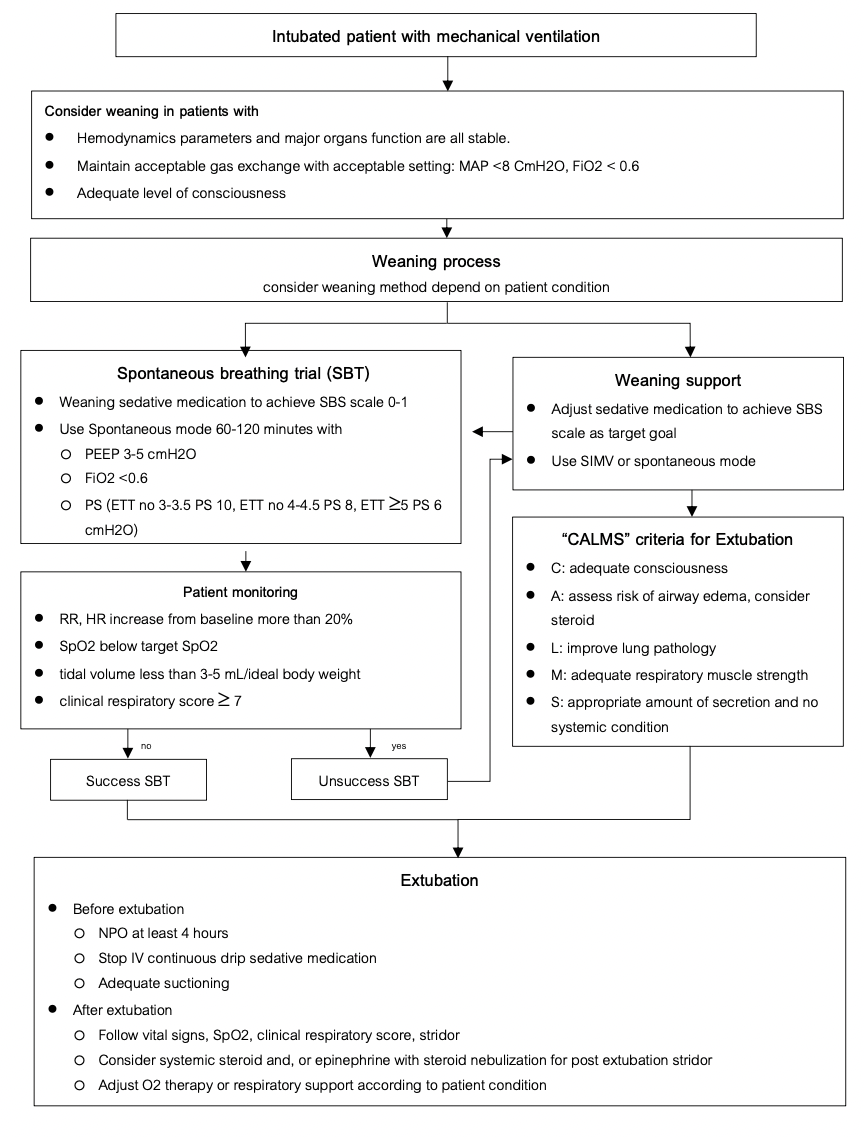


**Supplementary Figure 1.** Guideline for weaning and extubation readiness assessment.


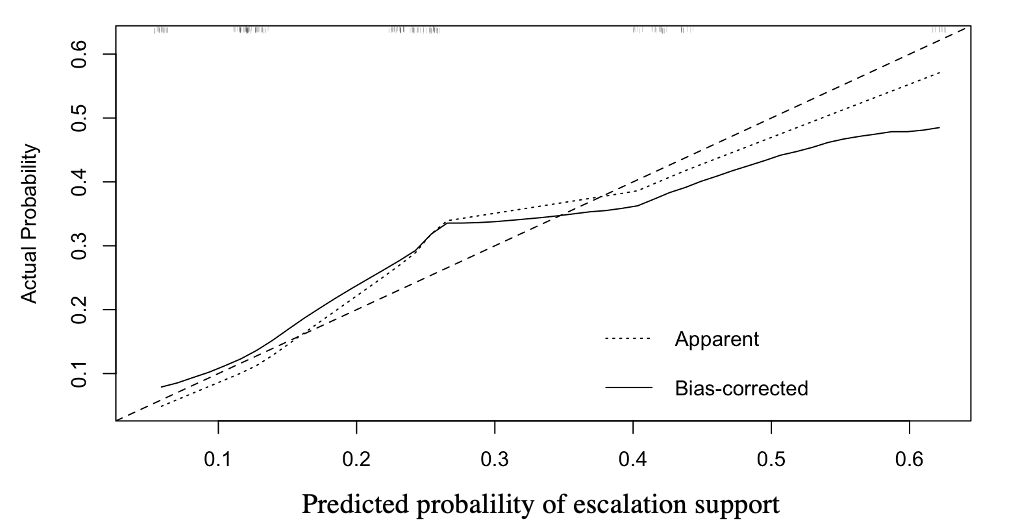


**Supplementary Figure 2.** Validation plot for the predictive model by bootstrap resampling method with a mean absolute error of 3.8.


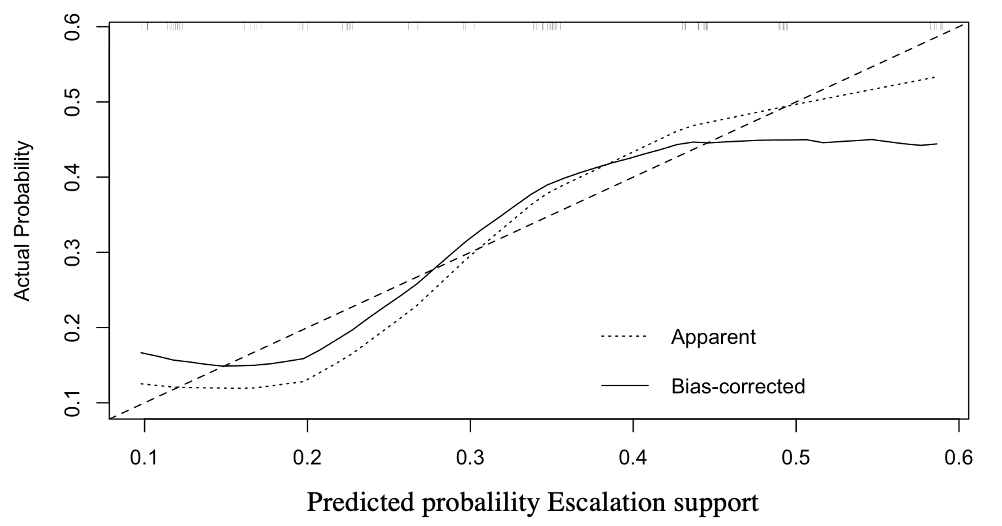


**Supplementary Figure 3.** Validation plot for the predictive model in postoperative patients by bootstrap resampling method with mean absolute error of 4.1.


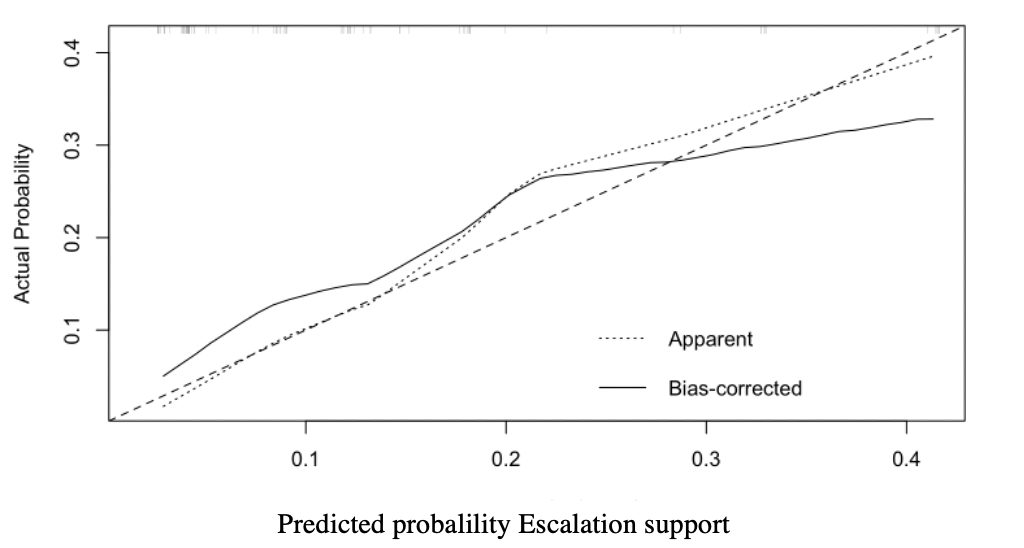


**Supplementary Figure 4.** Validation plot for the predictive model in medical patients using the bootstrap resampling method with mean absolute error 4.1.


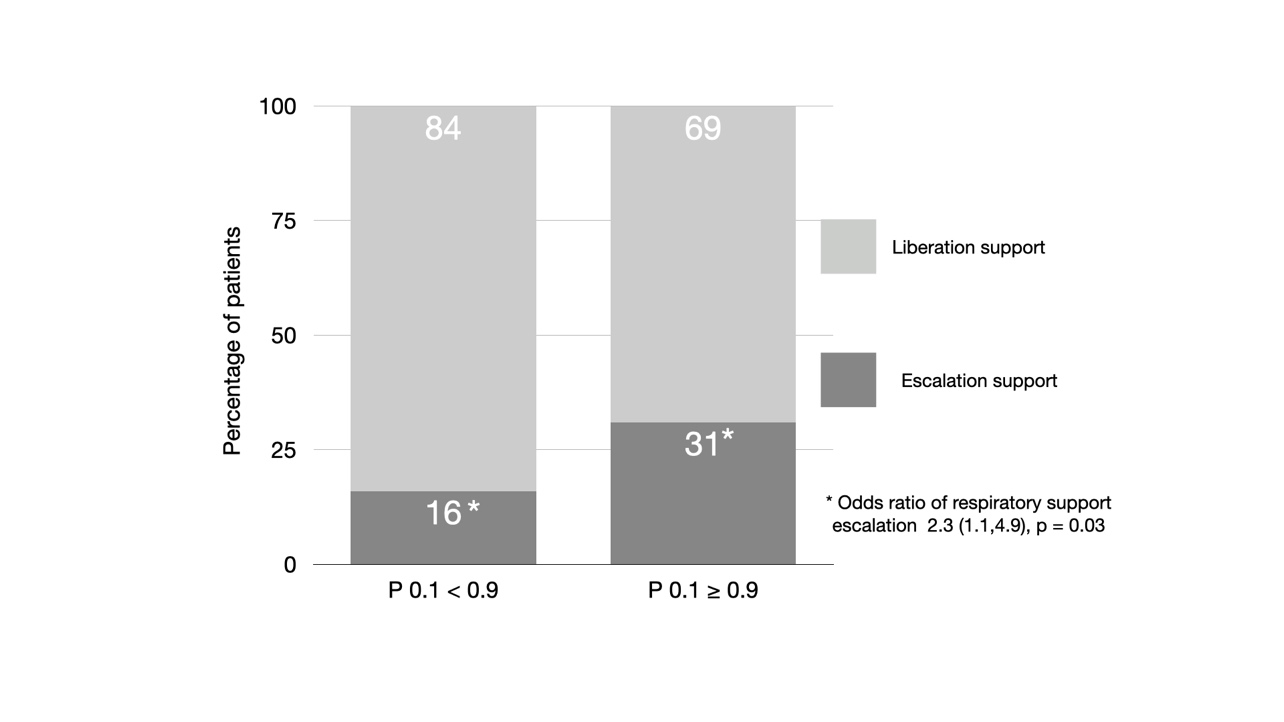


**Supplementary Figure 5** The distribution of P0.1 at 30 minutes after SBT. The Odds ratio of escalation respiratory support in patients who had P0.1 ≥0.9 after SBT was 2.3 (1.1, 4.9), p=0.03.

## Supplementary Tables

**Supplementary Table 1.** Ventilator parameters during the study period

|  | Escalation support  n = 45 (%) | Liberation support  n = 143 (%) | | *P* value | |  |
| --- | --- | --- | --- | --- | --- | --- |
| Non-minimal SBT | 24 (53.3) | 46 (32.2) | | 0.017 | |  |
| Pressure support (cmH_2_O) (median, IQR) | 8 (7,10) | 8 (7,10) | | 0.585 | |  |
| PEEP >5 cmH_2_O | 18 (40) | 31 (21.7) | | 0.025 | |  |
| **At 0 min (median, IQR)** | | | | | | |
| P0.1 (cmH_2_O) | 0.9 (0.7,1.3) | 0.8 (0.5,1.4) | | 0.173 | |  |
| RR (/min) | 28 (20,34) | 23 (19,32) | | 0.091 | |  |
| Vti/kg (mL/kg) | 9.2 (6.3,11.8) | 9.2 (7.2,11.8) | | 0.413 | |  |
| Vte/kg (mL/kg) | 8.2 (5.8,11.3) | 8.5 (6.6,10.8) | | 0.407 | |  |
| SpO_2_ | 100 (100,100) | 100 (99,100) | | 0.584 | |  |
| HR* (/min) | 117.3 (24.7) | 115.5 (27.2) | | 0.695 | |  |
| RSBI (breath/min/kg) | 304.9 (127.5,810.8) | 255.8 (104.6,603) | | 0.27 | |  |
| cRSBI (breath/min/mL/kg) | 30.3 (6.4,89.2) | 23.4 (5.8,101.2) | | 0.346 | |  |
| **At 30 min (median, IQR)** | | | | | | |
| P0.1 (cmH_2_O) | 1 (0.8,1.4) | 0.8 (0.5,1.4) | | 0.063 | |  |
| RR (/min) | 28 (20,36) | 24 (18,30) | | 0.063 | |  |
| Vti/kg (mL/kg) | 8.7 (7.3,10.9) | 9.8 (7.5,13) | | 0.121 | |  |
| Vte/kg (mL/kg) | 8 (6.8,9.7) | 9 (6.6,11.1) | | 0.084 | |  |
| SpO_2_ | 100 (100,100) | 100 (99,100) | | 0.862 | |  |
| HR* (/min) | 119.5 (24.7) | 114.2 (24) | | 0.198 | |  |
| **At 60 min (median, IQR)** | | | | | | |
| P0.1 (cmH_2_O) | 0.9 (0.7,1.5) | 0.9 (0.6,1.5) | | 0.424 | |  |
| RR (/min) | 28 (20,36) | 26 (19,32) | | 0.123 | |  |
| Vti/kg (mL/kg) | 8.3 (6.8,11.1) | 9.2 (6.8,12.2) | | 0.27 | |  |
| Vte/kg (mL/kg) | 7.6 (6.6,10.3) | 8.3 (6.4,10.8) | | 0.458 | |  |
| SpO_2_ | 100 (99,100) | 100 (100,100) | | 0.377 | |  |
| HR (/min) | 125 (106,134) | 119 (98,136) | | 0.54 | |  |
| **At 120 min (median, IQR)** | | |  | |  |  |
| P0.1 (cmH_2_O) | 0.9 (0.5,1.2) | 0.9 (0.5,1.3) | | 0.995 | |  |
| RR (/min) | 28 (18,38) | 24 (18,32.2) | | 0.144 | |  |
| Vti/kg (mL/kg) | 8.6 (5.8,11.2) | 9.5 (7.6,12) | | 0.049 | |  |
| Vte/kg (mL/kg) | 7.2 (5.1,10) | 8.8 (7,11.2) | | 0.009 | |  |
| SpO_2_ | 100 (98,100) | 100 (100,100) | | 0.054 | |  |
| HR* (/min) | 120.6 (27.3) | 115.4 (29) | | 0.291 | |  |
| **Before extubation (median, IQR)** | | | | |  |  |
| P0.1 (cmH_2_O) | 0.9 (0.7,1.9) | 1 (0.6,1.6) | | 0.692 | |  |
| RR (/min) | 27 (20,40) | 26 (19.2,33) | | 0.433 | |  |
| Vti/kg (mL/kg) | 9.2 (6.7,11.6) | 9.3 (7.3,12.5) | | 0.285 | |  |
| Vte/kg (mL/kg) | 8 (3.9) | 8.8 (3.7) | | 0.192 | |  |
| SpO_2_ | 100 (100,100) | 100 (100,100) | | 0.991 | |  |
| HR* (/min) | 121.6 (27.9) | 120.7 (27.1) | | 0.842 | |  |
| Delta Vti (mL/kg) | -0.3 (-1.6,0.5) | 0.1 (-1.1,1.8) | | 0.07 | |  |
| Delta Vte (mL/kg) | -0.8 (-2.2,0.7) | 0.2 (-0.9,1.5) | | 0.029 | |  |

* mean (±SD)

SBT, spontaneous breathing trial; IQR, interquartile range; PEEP, positive end-expiratory pressure; P0.1: occlusion pressure; RR, respiratory rate; Vti: inspired tidal volume; Vte: exhaled tidal volume; SpO_2_: oxygen saturation; HR, heart rate; RSBI: rapid shallow breathing index; cRSBI: corrected rapid shallow breathing index; Delta Vti: inspired tidal volume change between 120 and 0 min; Delta Vte: exhaled tidal volume change between 120 and 0 min

**Supplemental Table 2**. Initial respiratory support after extubation

| **Type of respiratory support** | **Escalation support**  **n = 45 (%)** | **Liberation support**  **n = 143 (%)** | ***P* value** 0.197 |
| --- | --- | --- | --- |
| Low flow oxygen | 20 (44.4) | 70 (49) |  |
| HFNC | 17 (37.8) | 62 (43.4) |  |
| BiPAP/NIPPV | 6 (13.3) | 9 (6.1) |  |
| CPAP | 0 (0) | 1 (0.7) |  |
| None | 1 (2.2) | 2 (1.4) |  |

HFNC, high-flow nasal cannula; BiPAP, bilevel positive airway pressure; NIPPV, noninvasive positive pressure ventilation; CPAP, continuous positive airway pressure

**Supplemental table 3** Multivariate analysis of predictors of respiratory support escalation from the cause other than upper airway obstruction

|  | **Odds ratio (95% CI)** | **P value** |
| --- | --- | --- |
| Nonminimal-setting SBT | 2.2 (1.1, 4.6) | 0.03 |
| > 3 ventilator days | 2.4 (1.2, 4.9) | 0.02 |
| P0.1 at 30 minutes ≥ 0.9 cmH_2_O | 2.2 (1.1, 4.8) | 0.03 |
| Vte/kg at 120 minutes ≤ 8 mL/kg | 2.2 (1.1, 4.6) | 0.03 |

SBT: spontaneous breathing trial, P0.1: occlusion pressure, Vte: exhaled tidal volume
